# Awareness and attitude of the public toward personalized medicine in Korea

**DOI:** 10.1371/journal.pone.0192856

**Published:** 2018-02-16

**Authors:** Iyn-Hyang Lee, Hye-Young Kang, Hae Sun Suh, Sukhyang Lee, Eun Sil Oh, Hotcherl Jeong

**Affiliations:** 1 College of Pharmacy, Yeungnam University, Gyeongbuk-do, South Korea; 2 College of Pharmacy, Yonsei University, Inchon, South Korea; 3 Yonsei Institute of Pharmaceutical Sciences, Yonsei University, Inchon, South Korea; 4 College of Pharmacy, Pusan National University, Busan, South Korea; 5 College of Pharmacy, Ajou University, Kyeonggi-do, South Korea; 6 Department of Pharmaceutical Medicine and Regulatory Sciences, Colleges of Medicine and Pharmacy, Yonsei University, Incheon, Korea; 7 Department of Clinical Pharmacology and Clinical Trials Center, Severance Hospital, Yonsei University Health System, Seoul, Korea; 8 College of Pharmacy, Ewha Womans University, Seoul, South Korea; Tel Aviv University, ISRAEL

## Abstract

**Objectives:**

As personalized medicine (PM) is expected to greatly improve health outcomes, efforts have recently been made for its clinical implementation in Korea. We aimed to evaluate public awareness and attitude regarding PM.

**Methods:**

We performed a self-administered questionnaire survey to 703 adults, who participated in the survey on a voluntary basis. The primary outcome measures included public knowledge, attitude, and acceptance of PM. We conducted multinomial multivariate logistic analysis for outcome variables with three response categories and performed multivariate logistic regression analyses for dichotomous outcome variables.

**Results:**

Only 28% of participants had knowledge that genetic factors can contribute to inter-individual variations in drug response and the definition of PM (199 out of 702). Higher family income was correlated with greater knowledge concerning PM (OR = 3.76, p = 0.034). A majority of respondents preferred integrated pharmacogenomic testing over drug-specific testing and agreed to inclusion of pharmacogenomic testing in the national health examination (64% and 77%, respectively), but only 51% were willing to pay for it.

**Discussion:**

Our results identify the urgent need for public education as well as the potential health disparities in access to PM. This study helps to frame policies for implementing PM in clinical practice.

## Introduction

The last decade featured an unprecedented pace of advancement in medical sciences driven by genomic technologies [1, 2]. The cost of sequencing has dropped sharply and thus the general public has easier access to genetic testing and identification of genomes. Society is more likely to realize the advantages of pharmacogenomics (PGx), a major driver of personalized medicine (PM). In cases which the potential benefits of PGx are realized and PM is routinely adopted in clinical practice, we expect a wide range of positive outcomes: (i) saving time and cost by increasing the efficiency of clinical trials in the drug development process; (ii) improving drug efficacy and safety in the short term; and (iii) improving health outcomes with better quality-of-life for patients in the long term [3–7]. Thus, many regulatory agencies in developed countries have promoted application of PGx data to drug development processes and implementing personalized medicine in clinical practice.

While these advancements are certain to significantly impact the practice of medicine, the safe, effective, and appropriate use of this knowledge and technology requires reforming healthcare systems and policies. Given the wide range of stakeholders involved in PGx, it is essential to include these various perspectives as new healthcare systems and policies are developed [8]. Also, this process will be influenced by various social and ethical issues. Such issues in turn are largely affected by national or ethnic beliefs, traditions, and values, and thus social acceptance could vary among countries. This underscores the importance of examining key issues, such as public awareness and attitude, and developing governmental strategy accordingly [9, 10].

Recently, efforts have been made to frame strategies for implementing PM into clinical practice in Korea. The Ministry of Food and Drug Safety (MFDS; formerly the Korean Food and Drug Administration) has provided an outline for an internal proposal in which the integrated PGx testing for all functionally identified and validated PGx variants needs to be developed and promoted to the public as part of the health examination within the government-run National Health Insurance (NHI) system (see Methods for the strategic details). In assessing the feasibility of the proposal, we became interested in examining the awareness and attitude of the general public toward PGx and PM in Korea. Whether the nationwide implementation system proposed by MFDS will be initiated, it is crucial for the public to develop a clear understanding of both the benefits and limitations of PM and to understand the psychosocial and ethical issues related to these new technologies, including privacy, genetic discrimination, and health disparities in access to PM.

Therefore, this study was conducted in order: (i) to explore public awareness and attitude toward PM; (ii) to explore public acceptance of PGx testing as part of the National Health Examination; and (iii) to identify practical strategies that can support the proposed national system for PM by defining and analyzing the current challenges.

## Materials and methods

### Study subjects and data sources

A self-administered questionnaire survey was distributed to 706 adults who visited community pharmacies or public healthcare centers between December 31, 2012 and January 14, 2013. We made a choice of our sample size as consistent with those of previous studies investigating similar subjects [11–15]. We chose 13 study sites featuring diverse of demographic and medical characteristics of respondents. Our study sites were comprised of four pharmacies that primarily fill prescriptions for outpatients from general hospitals, seven community pharmacies providing over-the-counter medications and filling prescriptions from nearby local clinics, and two community healthcare centers. All sites were located in the Seoul metropolitan area. Twelve educated data collectors recruited participants on a voluntary basis and required them to complete a paper questionnaire. We excluded healthcare professionals such as physicians, nurses, and pharmacists in our study. For data analysis, we included questionnaires in which more than 90% of questions were completed. The survey was approved by the Institutional Review Board of Yonsei University Health System (Approval code: 4-2012-0793). The review board determined that verbal consent was enough for this minimal-risk survey. We received verbal informed consent from respondents prior to participation in this study.

### Measurements

We developed a questionnaire to examine public awareness and several aspects of attitudes regarding PM using PGx information. To identify any ambiguity in questions, we conducted a pilot test prior to completing the final version of the questionnaire which comprised three domains: *public knowledge/awareness of personalized medicine*, *public attitude toward personalized medicine*, *and public acceptance of integrated pharmacogenomic testing as part of the national health examination*.

### Public knowledge/awareness of personalized medicine

The level of knowledge or awareness of PM was assessed using two questions with a binary -response of ‘yes’ or ‘no.’ First, the respondents were asked whether they were aware that drug responses, including the occurrence of adverse drug reactions (ADRs), can vary depending on the patient’s genotype. Second, they were asked whether they knew that PM could improve the effectiveness of treatment and/or decrease ADRs by allowing selection of the most appropriate drug and dosage based on the genotype of the individual.

### Public attitude toward personalized medicine

Social acceptance is an essential prerequisite for the implementation of PM in clinical practice. Therefore, we assessed the public attitude concerning PM by asking about their willingness to take a PGx test and their preference of test type between drug-specific PGx testing and integrated PGx testing. The question followed a short explanation of PGx testing and its application in PM to ensure that all respondents had a baseline level of knowledge regarding PGx testing prior to answering the question. We reasoned that respondents’ willingness to take a PGx test would reflect their positive attitude toward and trust in PM.

In the survey, respondents were asked to choose their preference for test type from two different types of PGx testing: drug-specific PGx testing and integrated PGx testing. Drug-specific PGx testing refers to a test that people take as part of their drug therapy for treatment of a specific disease. Each patient underwent a PGx testing to reveal genetic variants which could affect his/her responses to specific drug(s) and such PGx results can help guide to make a choice of drugs and dosage. Currently, this is common in clinical practice. On the other hand, integrated PGx testing is a one-time, alternative approach that identifies genetic variants affecting response to various drugs and can be taken when an individual is in good health. Integrated PGx testing can have the advantage of determining whether an individual has higher efficacy, or more side effects, to certain drugs, thus saving time in cases requiring rapid drug therapy decisions, as is often the case for many diseases. This approach also saves money by reducing unnecessary fees for multiple tests over one’s lifetime, as this single integrated PGx test is designed to identify all functionally validated genetic variants affecting drug response. This idea has been considered by MFDS in Korea and necessary infrastructures and relevant issues are being reviewed.

### Public acceptance of integrated pharmacogenomic testing as part of the national health examination

To assess public acceptance of adding the integrated PGx test to publicly funded health examinations, respondents were asked their opinions using a 5-point Likert-type scale: 1, ‘strongly disagree’; 2, ‘disagree’; 3, ‘neutral’; 4, ‘agree’; and 5, ‘strongly agree.’ In addition, they were asked about their willingness to pay for the test. Major public concerns about the routine use of PGx testing in clinical practice were also investigated. The list of public concerns was developed based on a literature review of prior studies [16].

### Data analysis

*Outcome variables* in this study included awareness, attitude, preference, acceptance of PM, and willingness-to-pay. First, awareness was assessed as three levels: (i) ‘fully aware’ includes the case in which respondents answered ‘yes’ to both questions for genetic contribution to drug response and defining PM; (ii) ‘partially aware’ is defined as the case in which respondents answered ‘yes’ to one of the two questions; and (iii) ‘unaware’ includes the case in which respondents answered ‘no’ to both questions. Second, concerning attitude toward PM, ‘positive attitude’ indicates that respondents were willing to take either type of PGx testing (i.e., drug-specific PGx test and integrated PGx test), and ‘negative attitude’ is defined as the unwillingness to take either PGx test. Third, the preferred option of PGx test was surveyed among respondents who exhibited a positive attitude concerning PM. Subsequently, the acceptance of incorporating the integrated PGx testing into the national health examination (i.e., NHI health examination, NHE) was measured using a 5-point Likert scale. Lastly, willingness-to-pay was dichotomously measured as ‘yes’ or ‘no’.

*Explanatory variables* included sex, age, education level, monthly family income, medication utilization in the past 3 months, experience of switching medicine due to the lack of efficacy or drug adverse events, opinion of the performance of NHI, and concerns about the routine use of PM in clinical practice. Medication utilization in the past 3 months was grouped into 3 categories: none, < 1 month, or ≥1 month. We dichotomized negative experiences of switching medications due to lack of efficacy or drug adverse events, i.e., ‘yes’ or ‘no.’ Attitude toward the NHI was considered ‘positive’ if respondents answered ‘strongly agree’ or ‘agree’ to the question concerning the contribution of NHI in public health improvement, or ‘negative’ otherwise. Each concern about the clinical application of PGx testing was transformed into a binary variable ‘yes’ or ‘no.’ When necessary, some outcome variables were used as explanatory variables for other outcome variables.

The proportions and mean scale values for outcome variables were calculated to examine the awareness and overall attitudes of the general public regarding PM. We performed regression analyses to identify factors influencing public awareness and attitude toward PM using each outcome measurement as a dependent variable. For dichotomous outcome variables including attitude, preference, acceptance, and willingness-to-pay, multivariate logistic regression analyses were carried out. Acceptance variables were dichotomized into two categories for logistic regression: a positive acceptance (‘strongly agree’ or ‘agree’) with a value of 1, and a neutral or a negative acceptance (‘neutral,’ ‘disagree,’ or ‘strongly disagree’) with a value of 0. For awareness outcome variables with three response categories, multinomial multivariate logistic analysis was performed. Additionally, we performed multivariate logistic regression analyses to examine the relationship between respondents’ characteristics and each of the concerns regarding clinical application of PGx testing. To check the goodness-of-fit for models, c-statistics and the Hosmer-Lemeshow test were utilized. Data analysis was performed using SAS 9.2 version (SAS Institute Inc., Cary, NC, USA). Statistical significance was taken at p<0.05.

## Results

### Characteristics of the participants

A total of 706 individuals completed the questionnaire. Three respondents who left two or more questions blank (i.e., less than 90% of the questionnaire was completed) were excluded, and the remaining 703 participants were used for data analysis. As shown in Table 1, survey participants included more women (58.6%) than men (41.4%). Participants were approximately evenly distributed by 10-year age groups. Sixty-eight percent of respondents had college or post-graduate education. About two-thirds of respondents exhibited positive view concerning the performance of NHI in public health improvement.

**Table 1:** Socio-demographic and health characteristics of the survey respondents.

Characteristics		Number of respondents	% of respondents
**Sex**	Men	291	41.4
	Women	412	58.6
**Age (years)**	(Mean ± SD)	45.6±15.2	-
	20–29	126	17.9
	30–39	138	19.6
	40–49	147	20.9
	50–59	157	22.3
	≥60	135	19.2
**Education level**	Postgraduate	65	9.3
	College	416	59.2
	High school	178	25.3
	Middle school	28	4.0
	Primary school or below	16	2.3
**Monthly family income**^**a**^ **(million KRW)**	<1.00	42	6.0
	1.00–2.99	209	29.7
	3.00–4.99	242	34.4
	5.00–6.99	130	18.5
	≥ 7.00	74	10.5
	Unanswered	6	0.9
**Self-assessed health status**	Very good	59	8.4
	Good	325	46.2
	Fair	227	32.3
	Poor	82	11.7
	Very poor	10	1.4
**Medication history** **in the past 3 months**	None	245	34.9
	<1 month	244	34.7
	≥1 month	214	30.4
**Experience of medicine switch due to lack of efficacy**	Yes	367	52.2
	No	336	47.8
**Experience of** **drug adverse events**	Yes	245	34.9
	No	458	65.2
**To the contribution of NHI in public health improvement**	Strongly agree	190	27.0
	Agree	326	46.4
	Neutral	153	21.8
	Disagree	26	3.7
	Strongly disagree	8	1.1

N = 703 respondents

SD = standard deviation; KRW = Korean currency, won; NHI = National Health Insurance.

^a^One US dollar equals approximately 1,100 Korean won in 2010s. The mean monthly family income in 2010 was 3.58 million KRW according to the 2010 Korea National Health and Nutrition Examination Survey.

### Awareness and attitudes regarding personalized medicine

Our survey included five items regarding awareness and attitudes of the public toward PM (Table 2). About two thirds of respondents were aware of PM. Most respondents were partially aware of the genetic contribution to drug response (36.3%), but had limited knowledge concerning the scope of PM. Nearly 90% of respondents showed a positive attitude toward PM and expressed willingness to take a PGx test. Of these, 64% preferred integrated PGx testing over drug-specific PGx testing. The majority of respondents (77%) responded positively to including integrated PGx testing as part of the NHI Health Examination (NHE). In contrast, only half were willing to pay for the integrated PGx testing. The ratio of respondents who were willing to pay for the integrated PGx testing was slightly increased from 51.4% to 55% among the positive-attitude respondents.

**Table 2:** Awareness and attitudes of the general public toward personalized medicine using pharmacogenomic information.

Variable		Number of respondents (%)
Awareness^a^ (N = 702)	Fully aware	199 (28.4%)
	Partially aware	267 (38.0%)
	*Aware of genetic contribution to drug response*	*255 (36*.*3%)*
	*Aware of what PM is*	*12 (1*.*7%)*
	Unaware	236 (33.6%)
Attitude^b^ (N = 700)	Positive	627 (89.6%)
	Negative	73 (10.4%)
Preferred option for PGx test (N = 627 who showed a positive attitude)	Drug-specific PGx test	228 (36.4%)
	Integrated PGx test	399 (63.6%)
Acceptance of the idea of incorporating the integrated PGx test into NHI health examination (N = 703)	Strongly agree (= 5)	247 (35.1%)
	Agree (= 4)	293 (41.7%)
	Neutral (= 3)	127 (18.1%)
	Disagree (= 2)	33 (4.7%)
	Strongly disagree (= 1)	3 (0.4%)
Willingness-to-pay for the integrated PGx test in NHI health examination (N = 703)	Yes	361 (51.4%)
	No	342 (48.7%)
Willingness-to-pay for the integrated PGx test in NHI health examination among the positive attitude^b^ respondents (N = 627)	Yes	345 (55.0%)
	No	282 (45.0%)

PM = personalized medicine; PGx = pharmacogenomics; NHI = National Health Insurance; SD = standard deviation

^a^‘Fully aware’ is the case in which the respondents answered ‘yes’ to both questions for genetic contribution to drug response and what PM is; ‘Partially aware’ is the case of answering ‘yes’ to one of the two questions; and ‘Unaware’ is the case of answering ‘no’ to both questions; one participant who did not answer the question was excluded from the analysis.

^b^‘Positive attitude’ indicates that the respondents are willing to take either of the two types of PGx testing (i.e., drug-specific PGx test or the integrated PGx test). ‘Negative attitude’ means unwillingness to take any PGx test; three participants who did not answer the question were excluded from the analysis.

**Fig 1:**
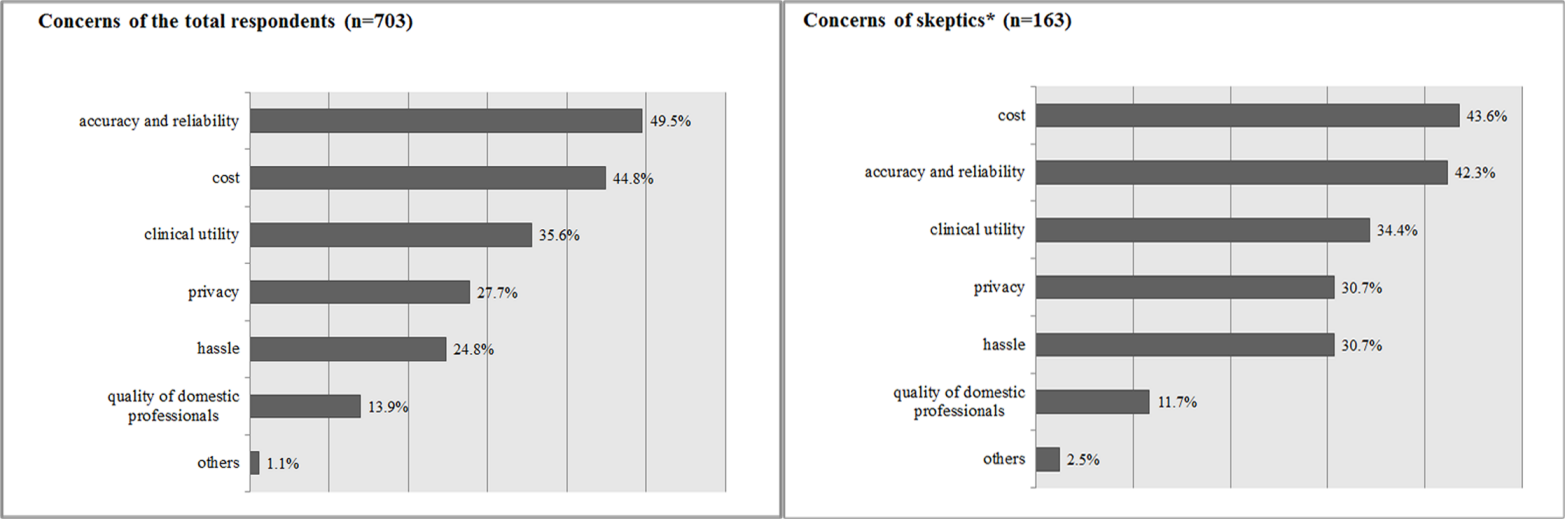
Concerns about clinical application of pharmacogenomic testing. Respondents were asked to select their two biggest concerns from a list. *Skeptics include those who were neutral, disagreed, or strongly disagreed with the idea of incorporating the integrated pharmacogenomics test into the National Health Insurance health examination.

Public concerns regarding PGx testing are shown in Fig 1. The accuracy/reliability of PGx testing was a top concern (49.5%), followed by cost (44.8%), clinical utility (35.6%), privacy (27.7%), and inconvenience (24.8%). A similar pattern of concerns was observed among the subgroup who gave a tepid response to the NHE idea.

### Factors associated with the public’s awareness and attitudes regarding personalized medicine

Multinomial multivariate logistic regression was performed for outcome variables of PM awareness (Table 3). The age group from 40–49 and the highest income group showed significantly greater odds of being aware of PM compared with lacking knowledge concerning PM (ORs = 2.02 and 3.76, respectively, p<0.05). No significant relationship was observed between other characteristics, i.e., sex, education, medication history and negative experiences with medicine, and awareness of PM.

**Table 3:** Multinomial multivariate logistic regression analysis results for factors that are associated with public awareness of personalized medicine in Korea.

Variable		Partially aware^a^ (vs. Unaware)		Fully aware^a^ (vs. Unaware)	
		adj. OR (95% CI)	*p* value	adj. OR (95% CI)	*p* value
**Sex**	Women (ref)				
	Men	0.73 (0.50–1.06)	0.098	0.79 (0.53–1.18)	0.251
**Age (years)**	20–29 (ref)				
	30–39	1.08 (0.61–1.91)	0.782	0.77 (0.40–1.47)	0.422
	40–49	1.31 (0.72–2.37)	0.380	2.02 (1.08–3.78)	0.028
	50–59	1.23 (0.68–2.23)	0.485	1.56 (0.83–2.95)	0.167
	≥60	1.09 (0.56–2.12)	0.799	1.54 (0.75–3.15)	0.236
**Education level**	Middle school or below (ref)				
	High school	1.70 (0.74–3.89)	0.211	1.00 (0.40–2.49)	0.999
	College or above	1.50 (0.65–3.48)	0.346	1.53 (0.62–3.78)	0.357
**Monthly family income**^**b**^ **(million KRW)**	<1.00 (ref)				
	1.00–2.99	0.75 (0.34–1.64)	0.473	1.70 (0.60–4.79)	0.319
	3.00–4.99	0.78 (0.35–1.78)	0.559	2.69 (0.93–7.75)	0.068
	5.00–6.99	0.80 (0.34–1.89)	0.609	1.65 (0.54–5.03)	0.381
	≥7.00	1.58 (0.58–4.29)	0.375	3.76 (1.11–12.81)	0.034
**Medication history in the past 3 months**	None (ref)				
	< 1 month	1.28 (0.83–1.99)	0.263	1.17 (0.73–1.87)	0.525
	≥1 month	0.91 (0.55–1.48)	0.692	0.92 (0.54–1.55)	0.746
**Negative experiences with medicines**	No (ref)				
	Yes	1.38 (0.95–2.01)	0.096	1.17 (0.78–1.76)	0.448

Ref = reference category; PM = personalized medicine; adj OR = adjusted Odds Ratio; KRW = Korean currency, won

^a^ ‘Fully aware’ is the case in which the respondents answered ‘yes’ to both questions for genetic contribution to drug response and what PM is; ‘Partially aware’ is the case of answering ‘yes’ to one of the two questions; and ‘Unaware’ is the case of answering ‘no’ to both questions; one participant who did not answer the question was excluded from the analysis.

^b^ One US dollar equals approximately to 1,100 Korean won in 2010s.

Multivariate logistic regression analyses were performed for outcome variables concerning the four aspects of public attitude (Table 4). Respondents choosing ‘accuracy and reliability of test,’ ‘privacy,’ and ‘quality of professionals’ as major concerns regarding PM were more likely to have a positive attitude toward PM (ORs = 2.95, 3.25 and 5.86, respectively, p<0.05). In addition, respondents with a positive view on the contribution of NHI in the improvement of public health tended to have a positive attitude toward PM (OR = 1.92, p = 0.021) and a preference for integrated PGx testing over drug-specific PGx testing (OR = 1.88, p = 0.002). A significant negative correlation was observed between men and a preference for integrated PGx testing over drug-specific testing (OR = 0.65, p = 0.018).

**Table 4:** Multivariate logistic regression analysis results for factors that are associated with public attitude toward personalized medicine in Korea.

Variable		adjusted OR (95% CI)			
		Positive attitude toward PM^a^ (n = 693^b^)	Integrated PGx testing (IPGT) preferred (n = 622^c^)	Acceptance for inclusion of IPGT into NHE (n = 693^b^)	Willingness to pay for IPGT (n = 693^b^)
**Sex**	Women (ref)				
	Men	0.85 (0.50–1.44)	0.65^†^ (0.50–0.93)	0.93 (0.62–1.38)	1.64^‡^ (1.16–2.32)
**Age (years)**	20–29 (ref)				
	30–39	1.71 (0.79–3.72)	0.98 (0.55–1.75)	1.12 (0.65–1.95)	0.54^†^ (0.31–0.92)
	40–49	1.55 (0.71–3.40)	0.77 (0.44–1.35)	1.52 (0.85–2.71)	0.63 (0.37–1.07)
	50–59	1.48 (0.65–3.33)	0.68 (0.38–1.22)	3.38^§^ (1.72–6.66)	0.60 (0.34–1.06)
	≥60	1.78 (0.68–4.63)	0.58 (0.30–1.12)	2.80^‡^ (1.30–6.03)	1.02 (0.53–1.97)
**Education level**	Middle school or below (ref)				
	High school	1.11 (0.32–3.84)	0.76 (0.34–1.69)	2.38 (0.92–6.17)	1.48 (0.66–3.32)
	College or above	0.73 (0.21–2.56)	0.99 (0.43–2.25)	1.47 (0.57–3.77)	1.49 (0.66–3.39)
**Monthly family income**^**d**^ **(million KRW)**	< 1.00 (ref)				
	1.00–2.99	0.69 (0.20–2.41)	0.88 (0.39–1.98)	0.97 (0.37–2.60)	3.49^‡^ (1.51–8.07)
	3.00–4.99	0.86 (0.24–3.13)	0.90 (0.39–2.08)	1.01 (0.37–2.76)	3.99^‡^ (1.68–9.46)
	5.00–6.99	0.72 (0.19–2.79)	0.76 (0.31–1.83)	1.08 (0.38–3.10)	4.18^‡^ (1.68–10.40)
	≥7.00	1.21 (0.26–5.71)	1.00 (0.38–2.68)	1.81 (0.56–5.87)	2.77^†^ (1.04–7.39)
**Medication history in the past 3 months**	None (ref)				
	<1 month	0.86 (0.47–1.56)	0.98 (0.65–1.48)	0.90 (0.58–1.41)	1.09 (0.73–1.62)
	≥1 month	1.08 (0.52–2.23)	1.25 (0.78–2.01)	1.12 (0.65–1.94)	1.31 (0.83–2.07)
**Negative experiences with medicines**	No (ref)				
	Yes	1.64 (0.96–2.79)	0.73 (0.51–1.06)	1.01 (0.67–1.52)	1.17 (0.82–1.66)
**Perception on the contribution of NHI in public health improvement**	Negative (ref)				
	Positive	1.92^†^ (1.10–3.35)	1.88^‡^ (1.26–2.80)	1.20 (0.78–1.86)	1.40 (0.95–2.05)
**Awareness of PM**	Unaware (ref)				
	Partially aware	0.74 (0.41–1.35)	0.93 (0.62–1.41)	1.16 (0.74–1.81)	1.52^†^ (1.02–2.26)
	Fully aware	1.06 (0.53–2.14)	0.74 (0.48–1.45)	1.60 (0.96–2.67)	2.05^§^ (1.33–3.16)
**Major concerns for clinical application of PGx testing**	Accuracy & reliability (yes vs. no)	2.95^†^ (1.10–7.87)	1.22 (0.52–2.90)	2.30 (0.99–5.38)	1.38 (0.57–3.32)
	Clinical utility (yes vs. no)	2.52 (0.90–7.01)	1.35 (0.55–3.28)	2.14 (0.90–5.11)	1.41 (0.57–3.48)
	Cost (yes vs.no)	2.42 (0.91–6.44)	1.35 (0.57–3.23)	2.23 (0.95–5.20)	1.11 (0.46–2.68)
	Hassle (yes vs.no)	1.17 (0.43–3.14)	0.91 (0.37–2.21)	1.51 (0.63–3.62)	1.06 (0.43–2.63)
	Privacy (yes vs.no)	3.25^†^ (1.08–9.75)	0.88 (0.36–2.16)	1.61 (0.66–3.95)	1.56 (0.62–3.91)
	Quality of professionals (yes vs.no)	5.86^†^ (1.49–22.97)	1.73 (0.67–4.49)	1.62 (0.62–4.27)	1.54 (0.59–4.02)
**Attitude toward PM**^**a**^	Negative (ref)	-	-		
	Positive	-	-	4.70^§^ (2.73–8.09)	3.96^§^ (2.04–7.71)
**Acceptance for inclusion of IPGT into NHE**	Negative (ref)	-	-	-	
	Positive	-	-	-	3.20^§^ (2.09–4.91)
**c-statistic**		0.70	0.63	0.72	0.73
***p* value of Hosmer and Lemeshow test**		0.8565	0.4878	0.1173	0.5966

Ref = reference category; OR = odds ratio; CI = confidence interval; PM = personalized medicine; PGx = pharmacogenomics; NHI = National Health Insurance Health; NHE = NHI Health Examination; KRW = Korean currency, won

†p<0.05.

‡p<0.01.

§p<0.001.

^a^‘Positive attitude’ indicates that the respondents are willing to take either of the two types of PGx testing (i.e., drug-specific PGx test or the integrated PGx test).‘Negative attitude’ means unwillingness to take any PGx test.

^b^Ten respondents were excluded from the analysis because six participants did not answer the question of family income; one did not answer the questions of awareness; three did not answer the question of the preferred type of testing.

^c^Among 627 respondents who were willing to take one of suggested PGx tests, five respondents were excluded from the analysis because four did not answer the question of family income; one did not answer the questions of awareness.

^d^One US dollar equals approximately 1,100 Korean won in 2010s.

The respondents aged 50–59, as well as 60 and above, were about 3 times more likely to accept the inclusion of integrated PGx test into the NHI national health examination compared to the respondents aged 20–29 (ORs = 3.38 and 2.80, respectively, p<0.01). Furthermore, a positive attitude toward PM (i.e., willingness to take a PGx test) was an additional factor associated with acceptance of integrated PGx testing (OR = 4.70, p<0.001).

Regarding the outcome variable of the willingness to pay for integrated PGx testing, a number of factors were identified to have a significant association. The odds for agreeing with an extra payment were 1.64 times greater for men than women (OR = 1.64, p = 0.005); 3~4 times greater for middle-income respondents (1–6.99 million KRW) than low-income respondents (less than 1 million KRW per month) (ORs = 3.49, 3.99 and 4.18, respectively, p<0.05); and 1.5~2 times greater for those aware of PM than those without prior knowledge (ORs = 1.52 and 2.05, respectively, p<0.05). Interestingly, the highest-income group (≥7 million KRW) exhibited the smallest OR (OR = 2.77, p = 0.042). Lastly, a positive attitude toward PM and the NHE proposal of including integrated PGx testing was strongly associated with the willingness to pay for PGx testing (OR = 3.96 and 3.20, respectively, p<0.001). Among the age groups, those aged 30–39 were less willing to pay for the test (OR = 0.54, p = 0.025).

### Major concerns related to clinical application of PGx testing

As shown in Table 5, multivariate logistic analyses suggest that senior respondents (≥50 years old) were more likely to be concerned with technological aspects, such as accuracy and reliability of testing or the quality of professionals who would perform testing procedures (ORs = 2.39~6.91, p<0.05). Educated respondents did not seem to mind the cost or potential inconvenience for testing (ORs = 0.26~0.45, p<0.05), but considered technological issues more important (OR = 2.41, p<0.05). Respondents who were aware of PM were less likely to be concerned with the cost (ORs = 0.46~0.58, p<0.01).

**Table 5:** Multivariate logistic regression analysis results for factors that are associated with public concerns about clinical application of pharmacogenomic testing in Korea.

Variable		adjusted OR (95% CI) n = 696^a^					
		Accuracy & reliability	Clinical utility	Cost	Inconvenience	Privacy	Quality of professionals
**Sex**	Women (ref)						
	Men	0.90 (0.66–1.25)	1.00 (0.72–1.39)	0.99 (0.72–1.38)	0.96 (0.66–1.39)	1.21 (0.85–1.73)	0.96 (0.60–1.54)
**Age (years)**	20–29 (ref)						
	30–39	1.19 (0.72–1.97)	1.22 (0.74–2.02)	0.81 (0.49–1.34)	0.67 (0.38–1.19)	1.15 (0.68–1.95)	1.13 (0.45–2.87)
	40–49	1.47 (0.89–2.41)	0.95 (0.58–1.58)	0.86 (0.52–1.42)	0.73 (0.41–1.28)	0.86 (0.50–1.46)	1.54 (0.64–3.69)
	50–59	2.39^§^ (1.43–3.98)	0.94 (0.56–1.57)	0.40^§^ (0.23–0.67)	0.58 (0.32–1.04)	0.89 (0.51–1.53)	3.45^‡^ (1.50–7.91)
	≥60	1.77 (1.00–3.15)	0.62 (0.34–1.14)	0.48^†^ (0.27–0.87)	1.00 (0.53–1.89)	0.41^†^ (0.21–0.82)	6.91^§^(2.85–16.73)
**Education level**	Middle school or below (ref)						
	High school	1.59 (0.76–3.29)	1.82 (0.76–4.35)	0.48 (0.23–1.02)	0.45^†^ (0.21–0.95)	1.28 (0.47–3.46)	2.98 (0.82–10.89)
	College or above	2.41^†^ (1.15–5.03)	1.69 (0.71–4.03)	0.26^§^ (0.12–0.56)	0.35^‡^ (0.16–0.74)	1.67 (0.63–4.48)	5.06^†^ (1.39–18.33)
**Monthly family income (million KRW** ^**b**^**)**	< 1.00 (ref)						
	1.00–2.99	1.19 (0.58–2.44)	0.95 (0.42–2.11)	0.91 (0.43–1.89)	1.29 (0.58–2.84)	0.69 (0.28–1.72)	1.61 (0.55–4.75)
	3.00–4.99	1.50 (0.71–3.16)	1.00 (0.44–2.27)	0.71 (0.33–1.52)	1.11 (0.48–2.55)	0.84 (0.33–2.09)	1.24 (0.40–3.85)
	5.00–6.99	1.00 (0.45–2.19)	1.09 (0.46–2.58)	0.91 (0.41–2.03)	1.11 (0.46–2.68)	0.93 (0.36–2.42)	1.69 (0.52–5.49)
	≥7.00	1.46 (0.61–3.48)	0.68 (0.26–1.76)	0.97 (0.40–2.35)	0.73 (0.26–2.05)	1.15 (0.42–3.19)	1.52 (0.42–5.48)
**Medication history in the past 3 months**	None (ref)						
	< 1 month	1.05 (0.72–1.51)	0.71 (0.49–1.04)	1.13 (0.78–1.65)	1.57^†^ (1.02–2.41)	0.79 (0.52–1.18)	1.14 (0.64–2.02)
	≥1 month	1.10 (0.72–1.68)	0.65 (0.42–1.00)	1.29 (0.84–1.99)	0.99 (0.60–1.64)	0.99 (0.62–1.58)	1.13 (0.62–2.06)
**Negative experiences with medicines**	No (ref)						
	Yes	0.73 (0.53–1.02)	1.10 (0.78–1.53)	0.93 (0.67–1.29)	0.93 (0.64–1.35)	1.29 (0.89–1.87)	1.30 (0.80–2.13)
**Perception on the contribution of NHI in public health improvement**	Negative (ref)						
	Positive	0.74 (0.51–1.05)	1.08 (0.75–1.56)	1.18 (0.82–1.70)	1.10 (0.73–1.67)	1.18 (0.79–1.77)	0.74 (0.44–1.27)
**Awareness of PM**	Unaware (ref)						
	Partially aware	0.89 (0.61–1.28)	1.22 (0.84–1.79)	0.58^‡^ (0.40–0.84)	1.14 (0.75–1.73)	1.31 (0.86–1.99)	1.52 (0.86–2.69)
	Fully aware	1.14 (0.77–1.70)	1.14 (0.76–1.73)	0.46^§^ (0.31–0.70)	0.92 (0.58–1.46)	1.49 (0.96–2.33)	1.61 (0.88–2.91)
**c-statistic**		0.62	0.61	0.65	0.63	0.64	0.70
***p* value of Hosmer and Lemeshow test**		0.1336	0.2319	0.2563	0.3739	0.1477	0.7816

Ref = reference category; OR = odds ratio; CI = confidence interval; PM = personalized medicine; NHI = National Health Insurance Health; KRW = Korean currency, won

†p<0.05.

‡p<0.01.

§p<0.001.

^a^Seven respondents were excluded from the analysis because six participants did not answer the question of family income; one did not answer the questions of awareness.

^b^One US dollar equal approximately 1,100 Korean won in 2010s.

## Discussion

This study explored public awareness and attitudes toward PM in Korea and evaluated the feasibility of an MFDS internal proposal for the implementation of PM into the national healthcare system. The survey results revealed some notable points as follows: Approximately 28% of participants were aware of PGx testing. Another group, comprised of 36% of participants, recognized that genetic factors can contribute to inter-individual variations in drug response, but were unaware of PGx testing. This low level of public awareness is quite distinguishable from the high level (80%) in the U.S. population [13]. This is in line with a previous report suggesting the lower level of public awareness for genetic testing among Asians compared with Americans [15]. This substantial difference could be the result of information on genetic testing has seldom been publicly promoted. The public has yet to experience relevant social issues such as regulatory issues surrounding direct-to-consumer genetic testing in Korea.

Second, we hardly discovered any noticeable association between the degree of awareness and the socio-demographic characteristics of the respondents such as sex, age, and education. Haga and colleagues reported that older individuals, women, those with a college education, and those with experiences of drug adverse events were more interested in PGx testing [13]. In contrast, multivariate adjustment in this study did not show statistically significant associations concerning most covariates. This could be due to the low rate of awareness in the population. Among age groups, only the 40-49-age group showed significantly greater odds of being fully aware of PM. A possible explanation could be that people in their 40s begin to pay more attention to their health than younger people and maintain more access to health-related information than older groups. Family income was positively correlated with PGx knowledge.

Third, a majority of respondents preferred to integrated PGx testing over drug-specific PGx testing (64% vs. 36%), and answered in support of the proposed policy that itemizes PGx testing as part of the national health examination list (77%). Fewer numbers of respondents (51% among all respondents and 55% among the positive attitude respondents) answered that they were willing to pay for the test, suggesting that public interest in PGx testing cannot be directly interpreted as being accepted for their use of testing. Interestingly, a 2010 survey of the Canadian public indicates the same level (51%) of willingness to pay for direct-to-consumer (DTC) genetic testing [17]. However, in a 2010 survey of Americans, 70–80% of respondents expressed the willingness to pay for DTC genetic testing [18]. Although both studies were for DTC genetic testing and the subject of our study was confined to PGx testing, these findings are comparable. Caulfield and McGuire pointed out that a moderate level of willingness to pay for the Canadian public might stem from the fact that Canadians have a high expectation that the public healthcare system would pay for PGx testing [19]. We speculate that this could be true for Koreans who have the benefit of a publicly funded healthcare system. In the present study, the willingness of Koreans to pay for PGx testing was dependent on their characteristics, such as high income, prior knowledge concerning PM, and attitude toward the National Healthcare Insurance system.

Furthermore, this study revealed that the public in general lacks confidence in recently developed technologies of PGx and its application to clinical practice and public health. This finding can be interpreted as either avoiding the financial burden of testing or inconvenience associated with taking a ‘test.’ Interestingly, educated people were more doubtful about technological advances in this field, supporting the results of a previous Finnish study that suggested that better knowledge might bring ‘skepticism’ as well as ‘enthusiasm’[20].

There are various limitations of the present study. First, as shown in the results, the public has limited familiarity with PGx testing and therefore, their attitudes toward PGx testing might be based on their partial knowledge. Therefore, this pilot study calls for follow-up studies as the public acquires greater knowledge concerning PM based on PGx testing. Second, our study sample of 703 respondents might be a limited representation of the national population, and thus study findings may feature limited generalizability. However, the respondents were diverse in terms of educational level and family income, and the demographic distribution was a good match with that of the latest survey for the Korea National Health and Nutrition Examination [21]. This may relieve the potential bias in the study findings.

In spite of these limitations, the present study is significant in that this is the first study of its kind in Korea. We hope that this could serve as a primer for the implementation of PM in clinical practice. The results of the survey indicate the urgent need for public education. Therefore, our study could provide guidance for public education of PM and other issues in making relevant policies. Although the Korean population features a low rate of illiteracy, the rate of health literacy has not been extensively studies. In order to improve the overall healthcare of the nation, it is important to explore the health literacy issue and incorporate contents of genomic medicine into public education. In addition, the education and training of healthcare professionals, especially primary healthcare providers, are crucial. Indeed, knowledge deficiencies among healthcare professionals impede the implementation of PGx in clinical practice [22]. In addition, this study suggests that policy makers should consider how to avoid disparities in access to the benefits of PM, as the willingness to pay is dependent on family income and possibly socio-economic status.

## Conclusions

The low level of public awareness regarding PM in Korea is likely associated with the lack of information as well as some individual socio-demographic characteristics. It is notable that family income showed a positive correlation with PGx knowledge. This highlights the urgent need for increasing public awareness as well as reducing potential health disparities in access to PM. This study will help to frame policies for implementing PM in clinical practice.
