# Correction: Awareness and attitude of the public toward personalized medicine in Korea

**DOI:** 10.1371/journal.pone.0195847

**Published:** 2018-04-09

**Authors:** Iyn-Hyang Lee, Hye-Young Kang, Hae Sun Suh, Sukhyang Lee, Eun Sil Oh, Hotcherl Jeong

The funding information is incomplete. The complete funding information is as follows: This study was supported by a grant (12182KFDA686) from the Korean Food and Drug Administration (now the Ministry of Food and Drug Safety) and the Yeungnam University Research Grant (215A580006). The contents of this article are solely the responsibility of the authors and do not necessarily represent the official views of the KFDA or Yeungnam University.
